# Racialized economic segregation, the built environment, and assault-related injury: Moderating role of green space and vacant housing

**DOI:** 10.1016/j.pmedr.2025.103128

**Published:** 2025-06-06

**Authors:** Lea A. Marineau, Kelly K. Jones, Amanda K. Small, Dennis W. Buckman, Mudia Uzzi, Melanie Sona, Erin I. Liedtke, Shannon N. Zenk

**Affiliations:** a National Institute on Minority Health and Health Disparities, 6707 Democracy Boulevard, Suite 800, Bethesda, MD 20892, USA; b National Institute of Nursing Research, 31 Center Drive, Bethesda, MD 20892, USA; c Information Management Services, Inc., 1455 Research Blvd, Suite 315, Rockville, MD, 20850, USA; d Johns Hopkins University Bloomberg School of Public Health, 615 N. Wolfe Street, Baltimore, MD 21205, USA

**Keywords:** Assault-related injury, Segregation, Built environment, Green space, Vacant housing

## Abstract

**Objective:**

To examine the association between racialized economic segregation and assault-related injury hospital visits in metropolitan neighborhoods spanning five American states, and whether this relationship varies by vacant housing and green space.

**Methods:**

Data on assault visits from 2016 to 2019 Healthcare Cost and Utilization Project were linked with population, landcover, and park data for Arizona, Florida, Georgia, North Carolina, and New York. Racialized economic segregation was operationalized using Index of Concentration at the Extremes (ICE) for household income by race. All assault and firearm assault visits were aggregated by ZIP Code Tabulation Area and discharge year. Negative binomial regression was used to calculate incident rate ratios (aIRR) to estimate associations between segregation and assaults. Interaction terms between segregation and each green space and vacant housing moderator were included in separate models, adjusting for state, year of discharge, and population demographics.

**Results:**

For each 1-SD increase in ICE (increased advantage), all assault and firearm assault visit rates decreased (aIRR 0.62[0.60,0.64] and aIRR 0.43[0.41,0.45], respectively). Higher levels of vacant housing were associated with more all assault visits in the most disadvantaged neighborhoods but not in advantaged neighborhoods. Higher levels of forest space were protective for all assault and firearm assault visits in the most disadvantaged neighborhoods but not in advantaged neighborhoods.

**Conclusions:**

Neighborhoods with the least economic and social resources had higher all assault and firearm assault visit rates than neighborhoods with more. Our results highlight the potential mitigating role of forest space on the relationship between segregation and assault hospital visits.

## Highlights

• Racialized economic segregation was associated with assault hospital visits.
• Forest space may mitigate the relationship between segregation and assaults.
• Policies directed at addressing structural factors for violence are needed.
• Concentrated investment in the most vulnerable neighborhoods should be prioritized.

## 1 Introduction

In the United States (U.S.), violence-related injuries and homicides have been leading causes of morbidity and mortality for decades (Centers for Disease Control and Prevention NCfIPaC, 2022). Homicide is currently the third leading cause of death among adolescents and adults aged 15–34 years, and rates are highest among men and Black people (Centers for Disease Control and Prevention NCfIPaC, 2022). Firearms are more lethal than other weapons (Braga et al., 2021) and are used in the majority of homicides in the U.S. (Centers for Disease Control and Prevention NCfIPaC, 2022), and nonfatal firearm assault injuries are more severe than stabbing or blunt force assault-related injuries (Braga et al., 2021). There are also stark racial disparities in the spatial distribution of violence (Knopov et al., 2019; Uzzi et al., 2023). Hyper-segregated Black neighborhoods experience the highest levels of violence (Knopov et al., 2019; Uzzi et al., 2023) due to factors rooted in structural racism that created and maintain racialized residential segregation (Larrabee Sonderlund et al., 2022; Massey and Fischer, 2000). Defined by Bailey et al. (Bailey et al., 2017), structural racism “refers to the totality of ways in which societies foster racial discrimination through mutually reinforcing systems of housing, education, employment, earnings, benefits, credit, media, health care, and criminal justice” (Bailey et al., 2017). Systematic and intentional historical and contemporary racist policies and practices in housing lending (“redlining”) and neighborhood disinvestment have resulted in the spatial concentration and marginalization of Black neighborhoods, restricting access to resources and economic mobility, and generating neighborhood poverty, social disadvantage, and housing instability – factors associated with violence (Uzzi et al., 2023; Larrabee Sonderlund et al., 2022; Massey and Fischer, 2000; Uzzi et al., 2024).

One of the pathways by which segregation creates racial health inequities is by negatively affecting the income of residents, resulting in economic segregation (Larrabee Sonderlund et al., 2022). Racialized economic segregation considers the interaction between racial segregation and economic segregation, and simultaneously reflects spatial and social segregation (Larrabee Sonderlund et al., 2022; Krieger et al., 2015). Racialized economic segregation is frequently operationalized using the Index of Concentration at the Extremes (ICE) for household income by race, measuring spatial concentrations of racial and economic advantage (e.g., high-income White households) versus disadvantage (e.g., low-income Black households) within a specified area (Larrabee Sonderlund et al., 2022; Krieger et al., 2015). An expanding body of literature has found racialized economic segregation is strongly associated with multiple adverse health outcomes (Larrabee Sonderlund et al., 2022) including violence-related injury (Krieger et al., 2017; Jay et al., 2022).

The built environment (e.g., “human-made or -modified physical surroundings in which people live, work, travel, and play” (Cheadle and A., 2014)) is shaped by segregation and includes abandoned and vacant housing and green space (Bailey et al., 2017). Disinvestment in low-income, Black neighborhoods has resulted in more abandoned buildings and vacant houses (Branas et al., 2013; Roude et al., 2024), less green space (Jesdale et al., 2013; Wen et al., 2013), and poorer quality green space (Rigolon, 2016) compared to higher-income White neighborhoods.

The presence of abandoned and vacant houses in low-income, Black neighborhoods is strongly associated with violent injuries (Branas et al., 2013). Vacant houses are physical manifestations of widespread neighborhood disinvestment and neglect that result from structural factors, such as limited access to economic opportunities, that drive violence (Uzzi et al., 2023; Uzzi et al., 2024). Additionally, their presence can contribute to decreased perceptions of safety, which may result in stigma and reduced social connectedness among neighbors (Roude et al., 2024; Garvin et al., 2013), signaling an area lacks social control, and potentially attracting illegal activity (MacDonald, 2015).

In contrast, extant research has supported the protective effects of green space on violence outcomes. Green space, in the form of parks or greened lots, may decrease violence by encouraging positive street activity (e.g., walking, biking, social interactions) and, in turn, strengthening neighborhood social cohesion (Rupp et al., 2020; Heinze et al., 2018). More specifically, increased positive street activity can promote community connectedness through positive social interactions, which may improve neighbors' sense of responsibility, increasing neighborhood social control (Heinze et al., 2018; Aiyer et al., 2015).

Limited research has examined how to address the role of segregation on assault-related injuries. Given the evidence on the role of green space and vacant housing on violence, the harmful impact of segregation on assault-related injuries may be mitigated by green space and exacerbated by vacant housing. This can inform the development and placement of violence interventions targeting the built environment. The purpose of this study is to examine the association between racialized economic segregation and assault-related injury hospital visits in metropolitan neighborhoods spanning five U.S. states (Arizona, Florida, Georgia, North Carolina, New York); and whether this relationship varies by vacant housing and green space. Although not generalizable to the U.S., these five states are included because they represent different U.S. regions. We also include both all assault and firearm assault hospital visits in this analysis because firearm assaults are more lethal and may be associated with more harms compared to other assault-related injuries (Braga et al., 2021), and the relationship between them and the environmental context may differ.

## 2 Methods

For this serial cross-sectional study, metropolitan neighborhoods (proxied by ZIP Code Tabulation Areas (ZCTAs)) by year of discharge (2016–2019) were the unit of analysis (*N* = 12,648). U.S. Department of Agriculture Rural-Urban Commuting Area (RUCA) codes were used to identify metropolitan ZCTAs (United States Department of Agriculture, Economic Research Service, 2010). To link the ZIP Code-level RUCA codes with our ZCTA-level data, we used a 2018 ZIP to ZCTA crosswalk (John Snow, Inc, n.d.). ZCTAs are generalized areal representations of ZIP Codes (USC, n.d.).

Racialized economic segregation was operationalized using the ICE for household income by race (Krieger et al., 2015; Krieger et al., 2017) based on five-year Census data (2011–2015) from the American Community Survey (ACS) (U.S. Census Bureau, 2015-2019). It was calculated as the number of non-Hispanic White households with high incomes ($100,000/year or more) minus the number of non-Hispanic Black households with poverty-level incomes ($24,000/year or less), divided by total households in each. ICE ranges from −1 to +1, where −1 indicates ZCTAs with the greatest disadvantage and + 1 indicates ZCTAs with the greatest advantage (Krieger et al., 2015; Krieger et al., 2017). For ease of interpretation, we rescaled ICE by standard deviation and centered at the sample mean for this analysis. ZCTAs for which ICE could not be calculated were removed from the dataset (e.g., ZCTAs with zero population & ZCTAs missing number of households; *n* = 412).

Our moderators included vacant housing and green space. Vacant housing was measured as the percentage of vacant housing units within each ZCTA using 2011–2015 ACS data (U.S. Census Bureau, 2015-2019; Walker and Herman, 2024). To measure green space, we included three variables: (1) percentage of developed open space and (2) percentage of forest space within each ZCTA using the 2015 U.S. Geological Survey's National Land Cover Database (NLCD) (Dewitz, 2019) aggregated to the ZCTA-level by the National Neighborhood Data Archive (NaNDA) (Melendez et al., 2025), and (3) percentage of park area within each ZCTA compiled by the Trust for Public Land (Trust for Public Land, n.d.) and NaNDA (Li et al., 2020). Developed open space is one of 16 classifications of land cover derived from the NLCD Landsat satellite imagery at 30-m spatial resolution and includes private and public green spaces. It contains areas with a mixture of constructed materials, but mostly vegetation in the form of lawn grasses. Impervious surfaces (paved surfaces) account for less than 20 % of the total cover for developed open spaces, and developed open space is usually comprised of large-lot single family housing units, parks, golf courses, and vegetation planted in developed settings for recreation, erosion control, or aesthetic purposes (Dewitz, 2019). For forests, we combined 2015 NLCD classifications for deciduous forest, evergreen forest, and mixed forest. The forest measure is the percentage of ZCTAs that are dominated by trees that are greater than five meters tall, and greater than 20 % of the total vegetation cover (Dewitz, 2019). Park space comprises publicly owned local, state, and national parks; certain school parks; and privately owned parks that are publicly accessible. Parks in gated communities, golf courses, and private cemeteries are excluded from our park measure (Trust for Public Land, n.d.). For ease of interpretation, our vacant housing and green space variables were scaled such that each unit represents a ten-percentage point change. Appendix Table 1 shows the correlation matrix of our built environment measures (vacant housing and green space). They are all weakly correlated (range *r* = −0.296 to *r* = 0.235). Additionally, the correlations support the idea that the green space variables are measuring different aspects of green space.

Two outcomes were examined: (1) all assault-related injury (firearm, stabbing, blunt force) hospital visits, and (2) firearm assault-related injury hospital visits. The data were obtained from the 2016–2019 Agency for Healthcare Research and Quality Healthcare Cost and Utilization Project's (HCUP) State Emergency Department Database (SEDD) for emergency department discharges and State Inpatient Database (SID) for hospital admissions (HCUP State Emergency Department Databases (SEDD), 2016-2019; HCUP State Inpatient Databases (SID), 2016-2019). Data from 2016 to 2019 were chosen because of changes in hospital visit patterns during the COVID-19 pandemic, which began in 2020. SEDD and SID data include all emergency department discharges and inpatient hospitalizations for all or nearly all community hospitals in each state, excluding federal hospitals (e.g., Veterans Affairs Hospitals, Indian Health Service) (HCUP State Emergency Department Databases (SEDD), 2016-2019; HCUP State Inpatient Databases (SID), 2016-2019). The assault-related injury codes were based on the International Classification of Diseases, 10th Revision, Clinical Modification (ICD-10-CM) codes for external causes of injury (ICD-10-CM codes: Blunt force Y00, Y04, Stabbing X99, Firearm X93, X94, X95) (World Health Organization, 2004; Hedegaard et al., 2020). People identified as unhoused or who did not have a home ZIP Code were removed from the dataset. We also removed people whose residential state did not match the hospital state (e.g., a person from Arizona is admitted for an assault in Florida). We aggregated all assault- and firearm assault-related injury hospital visits by ZCTA and year of discharge. Emergency department discharges or hospital admissions with more than one type of assault (e.g., a person admitted for a stabbing and blunt assault) were counted once. The aggregated all assault- and firearm assault-related injury hospital visit counts (hereafter ‘all assault visits’ & ‘firearm assault visits’) from the HCUP SEDD and SID were linked with the 2011–2015 ACS, 2015 NLCD, and 2018 park data.

Included covariates were year of discharge, state, and four ZCTA-level variables that are known confounders: (1) population density, (2) population low educational attainment (below high school or General Educational Development), (3) population median age, and (4) population percentage male. Population data were obtained from the 2011–2015 ACS (U.S. Census Bureau, 2015-2019).

### 2.1 Statistical analysis

We calculated population weighted descriptive statistics for the study variables by state. Segregation ICE scores were not standardized when measuring their population weighted mean by state. ZCTA-level median age was missing from 56 ZCTAs (0.4 %), therefore we performed complete case analysis omitting those ZCTAs. We used negative binomial regression analysis to calculate adjusted incident rate ratios (aIRR) to estimate associations between standardized segregation ICE scores and assault visits (all assaults and firearm assaults separately) because assault visits were overdispersed and right-skewed counts (Gardner et al., 1995). We included logged, ZCTA-level population as the offset. Interaction terms between segregation ICE scores and each moderator (three green space variables and vacant housing) were included in separate models, adjusting for each other, state, year of injury, population density, median age, educational attainment, and percentage male. The standard errors are cluster robust and account for repeated measures on each ZCTA. Predictive margins were used to estimate the average predicted count of all assault visits and firearm assault visits, at discrete levels of segregation ICE scores by discrete levels of each significant moderator, adjusting for green space variables, vacant housing, state, year of injury, population density, median age, educational attainment, and percentage male (Graubard and Korn, 1999). The predictive margins (e.g., predicted marginal counts) were then plotted for visualization. We considered *P* values <0.05 statistically significant and all tests were two-tailed. The National Institutes of Health Institutional Review Board (IRB) determined that this study is not human subjects research, therefore IRB review and approval was not required. Retrieval of Census data was conducted using R tidycensus package (Walker and Herman, 2024) and analyses were conducted using Stata 18.0 (StataCorp, n.d.).

## 3 Results

Table 1 shows population weighted descriptive statistics of the study variables by state. New York had the highest annual mean all assault visits per ZCTA (population-weighted mean = 157 assault-related hospital visits) and North Carolina had the lowest (population-weighted mean = 78 assault-related hospital visits).

**Table 1:** Descriptive statistics of study variables by state.

	State					
	Arizona n(%)/ ZCTA mean^a^	Florida n(%)/ ZCTA mean^a^	Georgia n(%)/ ZCTA mean^a^	North Carolina n(%)/ ZCTA mean^a^	New York n(%)/ ZCTA mean^a^	Total n(%)/ ZCTA mean^a^
Metropolitan ZCTAs	956 (7)	3528 (28)	1864 (15)	1796 (14)	4560 (36)	12,704 (100)
ICE for Race & Income	0.13	0.08	0.05	0.07	0.14	0.10
Annual all assault-related injuries	92	79	82	78	157	108
Annual firearm assault-related injuries	5	4	5	4	4	4
Population density (square mile)	2663	2686	1250	795	22,619	9275
Median age in years	33	40	30	29	35	35
Low educational attainment rate per 100 residents^b^	13	13	11	10	14	12
Percentage male	44	47	41	37	44	43
Percentage vacant housing	12	16	10	8	8	11
Percentage developed open space	8	17	15	14	10	13
Percentage park space	9	5	3	2	7	5
Percentage forest space	1	7	30	27	12	14

No ZCTAs were omitted from the descriptive statistics.

GED, General Educational Development; ICE, Index of Concentration at the Extremes; ZCTA, ZIP Code Tabulation Area.

a Weighted by ZCTA population.

b Low educational attainment defined as less than high school or GED.

**Fig. 1:**
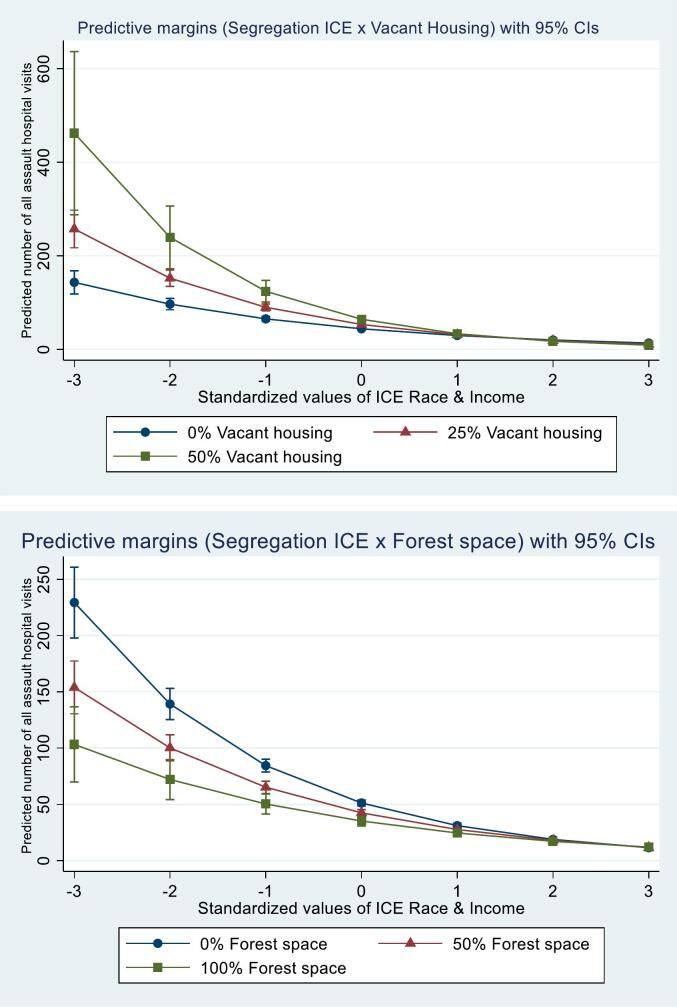
Predicted marginal count of 2016–2019 All assault-related injury hospital visits from metropolitan ZIP Code Tabulation Areas in Arizona, Florida, Georgia, North Carolina, and New York (*N* = 12,648). ICE, Index of Concentration at the Extremes.

Table 2 shows adjusted regression model results for all assault hospital visits. Model 1 is the adjusted main effects model. For each standard deviation increase in segregation ICE scores (increase in advantage), all assault visit rates decreased by 38 % (aIRR 0.62, 95 % CI 0.60, 0.64). For each ten-percentage point increase in vacant housing, developed open space, and park space, all assault visit rates increased by 8 %, 2 %, and 2 %, respectively (vacant housing: aIRR 1.08, 95 % CI 1.05, 1.11; developed open space: aIRR 1.02, 95 % CI 1.00, 1.04; park space: aIRR 1.02, 95 % CI 1.00, 1.04). For each ten-percentage point increase in forest space, all assault visit rates decreased by 4 % (aIRR 0.96, 95 % CI 0.95, 0.98). Model 6 is the final adjusted model including the only two significant interaction terms. Forest space and vacant housing moderated the relationship between segregation and all assault visits (segregation ICE x forest space aIRR 1.01, 95 % CI 1.00, 1.02 & segregation ICE x vacant housing aIRR 0.95, 95 % CI 0.92, 0.98). Fig. 1 shows a visualization of the predicted marginal count of all assault visits at discrete levels of segregation ICE scores by discrete levels of forest space and vacant housing, adjusting for all moderators, segregation ICE scores, significant interaction terms, state, year of injury, and ZCTA-level covariates.

**Table 2:** Negative binomial regression between Index of Concentration at the Extremes for household income by race and 2016–2019 All assault-related injury hospitalizations/emergency department discharges from metropolitan ZIP Code Tabulation Areas in Arizona, Florida, Georgia, North Carolina, and New York (N = 12,648).

Variables	Model 1	Model 2	Model 3	Model 4	Model 5	Model 6
	aIRR (95 % CI)	aIRR (95 % CI)	aIRR (95 % CI)	aIRR (95 % CI)	aIRR (95 % CI)	aIRR (95 % CI)
ICE Race & Income	0.62 (0.60, 0.64)	0.67 (0.64, 0.71)	0.63 (0.60, 0.67)	0.62 (0.60, 0.64)	0.61 (0.58, 0.63)	0.66 (0.62, 0.69)
Vacant houses	1.06 (1.04, 1.09)	1.08 (1.05, 1.11)	1.06 (1.04, 1.09)	1.06 (1.04, 1.09)	1.06 (1.04, 1.09)	1.08 (1.05, 1.11)
Developed open space	1.02 (1.00, 1.05)	1.02 (0.99, 1.04)	1.03 (1.00, 1.05)	1.02 (1.00, 1.05)	1.02 (1.00, 1.05)	1.02 (0.99, 1.04)
Park space	1.02 (1.00, 1.04)	1.02 (1.01, 1.04)	1.02 (1.00, 1.04)	1.02 (1.00, 1.04)	1.02 (1.00, 1.04)	1.02 (1.00, 1.04)
Forest space	0.96 (0.95, 0.98)	0.96 (0.95, 0.98)	0.96 (0.95, 0.98)	0.96 (0.95, 0.98)	0.96 (0.95, 0.98)	0.96 (0.95, 0.98)
Interaction terms	–	–	–	–	–	–
ICE Race & Income x vacant housing	–	0.95 (0.92, 0.97)	–	–	–	0.95 (0.92, 0.98)
ICE Race & Income x developed open space	–	–	0.99 (0.97, 1.01)	–	–	–
ICE Race & Income x park space	–	–	–	1.00 (0.98, 1.02)	–	–
ICE Race & Income x forest space	–	–	–	–	1.01 (1.00, 1.02)	1.01 (1.00, 1.02)

ICE Race & Income is scaled such that a one-unit change represents one-standard deviation and an increase in ICE score represents an increase in neighborhood advantage.

Vacant houses, Developed open space, Park space, and Forest space are the percentage of ZIP Code Tabulation Area they cover and are scaled such that a one-unit change represents ten-percentage points.

Models 1–6 (fully adjusted: year of discharge, state, population density, population low educational attainment (below high school or GED), population median age, and population percentage male).

aIRR, Adjusted incident rate ratio; GED, General Educational Development; ICE, Index of Concentration at the Extremes.

**Fig. 2:**
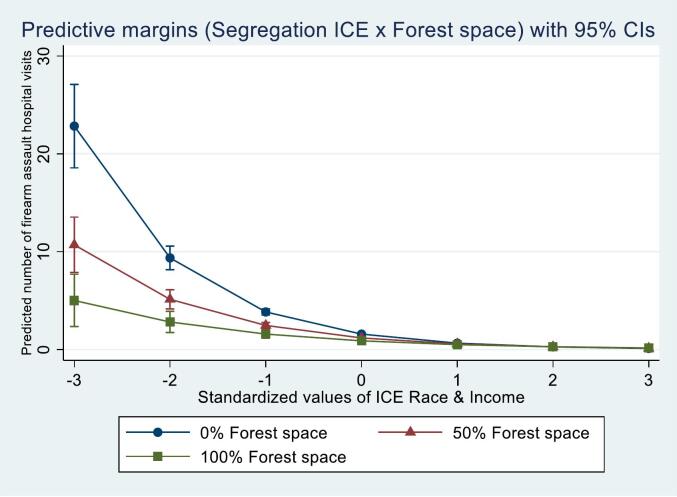
Predicted marginal count of 2016–2019 Firearm assault-related injury hospital visits from metropolitan ZIP Code Tabulation Areas in Arizona, Florida, Georgia, North Carolina, and New York (N = 12,648). ICE, Index of Concentration at the Extremes.

Table 3 shows adjusted regression model results for firearm assault hospital visits. Model 1 is the adjusted main effects model. For each standard deviation increase in segregation ICE scores (increase in advantage), firearm assault visit rates decreased by 57 % (aIRR 0.43, 95 % CI 0.41, 0.45). For each ten-percentage point increase in vacant housing and developed open space, firearm assault visit rates increased by 8 % and 8 %, respectively (vacant housing: aIRR 1.08, 95 % CI 1.03, 1.14; developed open space: aIRR 1.08, 95 % CI 1.04, 1.13). For each ten-percentage point increase in forest space, firearm assault visit rates decreased by 6 % (aIRR 0.94, 95 % CI 0.92, 0.96). Model 5 is the final adjusted model including the only significant interaction. Forest space (segregation ICE x forest space aIRR 1.03, 95 % CI 1.01, 1.05) moderated the relationship between segregation and firearm assault visits. Fig. 2 shows a visualization of the predicted marginal count of firearm assault visits at discrete levels of segregation ICE scores by discrete levels of forest space, adjusting for all moderators, segregation ICE scores, the significant interaction term, state, year of injury, and ZCTA-level covariates.

**Table 3:** Negative binomial regression between Index of Concentration at the Extremes for household income by race and 2016–2019 Firearm assault-related injury hospitalizations/emergency department discharges from metropolitan ZIP Code Tabulation Areas in Arizona, Florida, Georgia, North Carolina, and New York (N = 12,648).

Variables	Model 1	Model 2	Model 3	Model 4	Model 5
	aIRR (95 % CI)	aIRR (95 % CI)	aIRR (95 % CI)	aIRR (95 % CI)	aIRR (95 % CI)
ICE Race & Income	0.43 (0.41, 0.45)	0.44 (0.40, 0.49)	0.42 (0.39, 0.45)	0.43 (0.41, 0.45)	0.41 (0.38, 0.44)
Vacant houses	1.08 (1.03, 1.14)	1.08 (1.03, 1.14)	1.09 (1.03, 1.15)	1.08 (1.03, 1.14)	1.09 (1.03, 1.15)
Developed open space	1.08 (1.04, 1.13)	1.08 (1.04, 1.13)	1.09 (1.05, 1.13)	1.08 (1.04, 1.13)	1.08 (1.04, 1.13)
Park space	1.02 (0.98, 1.06)	1.02 (0.98, 1.07)	1.02 (0.98, 1.07)	1.02 (0.98, 1.06)	1.02 (0.98, 1.06)
Forest space	0.94 (0.92, 0.96)	0.94 (0.92, 0.96)	0.94 (0.92, 0.96)	0.94 (0.92, 0.96)	0.94 (0.92, 0.97)
Interaction terms	–	–	–	–	–
ICE Race & Income x vacant properties	–	0.98 (0.93, 1.04)	–	–	–
ICE Race & Income x developed open space	–	–	1.01 (0.98, 1.05)	–	–
ICE Race & Income x park space	–	–	–	1.01 (0.97, 1.05)	–
ICE Race & Income x forest space	–	–	–	–	1.03 (1.01, 1.05)

ICE Race & Income is scaled such that a one-unit change represents one-standard deviation and an increase in ICE score represents an increase in neighborhood advantage.

Vacant houses, Developed open space, Park space, and Forest space are the percentage of ZIP Code Tabulation Area they cover and are scaled such that a one-unit change represents ten-percentage points.

Models 1–5 (fully adjusted: year of discharge, state, population density, population low educational attainment (below high school or GED), population median age, and population percentage male).

aIRR, Adjusted incident rate ratio; GED, General Educational Development; ICE, Index of Concentration at the Extremes.

## 4 Discussion

Consistent with prior research, this study finds racialized economic segregation in metropolitan neighborhoods is strongly associated with violence outcomes (Krieger et al., 2017; Jay et al., 2022). This study extends prior research by finding this relationship varied by forest space and vacant housing. Higher levels of forest space were protective for all assault and firearm assault hospital visits in the most disadvantaged neighborhoods yet had little impact in the most advantaged neighborhoods. This aligns with prior research that found a greening intervention to have the largest firearm violence reductions in the highest-poverty neighborhoods (Branas et al., 2018).

Forest space was the only green space measure found to mitigate the relationship between segregation and assault hospital visits. Our study suggests the presence of dense concentrations of trees seen in forest space, and more ‘greenness’, has a more important role in violent injury reduction in the most disadvantaged neighborhoods, than the presence of parks or developed open space alone. Residents in disadvantaged neighborhoods may be more sensitive to the benefits of forest space. One study found vacant lot greening had the largest reductions in depression symptoms in the poorest neighborhoods (South et al., 2018). Exposure to natural environments, especially green space, reduces psychological and physiological stress (Yao et al., 2021; Kondo et al., 2018a). Forest space provides larger, concentrated doses of green space, and may offer more pleasant multisensory stimuli (Hedblom et al., 2019), which may be more restorative and calming than other types of green space, especially in neighborhoods experiencing more disadvantage.

Green space is a modifiable factor that can be targeted to reduce assaults in the most disadvantaged communities (Branas et al., 2018). This alone will not eliminate violence, but it is a low-cost intervention with promising results (Branas et al., 2018; Branas et al., 2016; Kondo et al., 2018b) that can offer co-benefits with other health, wellbeing, environmental, and social outcomes (Hunter et al., 2019). Future research should further examine different types of green space, including forest space, and their effects on violence in segregated neighborhoods, and possible mechanisms for these relationships.

Many complex, intersecting processes contribute to the observed relationship between racialized economic segregation and violence. Exposure to racialized economic segregation results from structural forces that maintain and protect advantage in majority-White neighborhoods by pushing racially minoritized populations into persistently deprived and low-income neighborhoods (Larrabee Sonderlund et al., 2022). Light and Thomas (2019) found racial segregation in metropolitan areas decreased the risk of homicide victimization among White people while simultaneously increasing the risk of homicide victimization among Black people (Light and Thomas, 2019), and their study did not consider racial and economic segregation together (racialized economic segregation) which has been found to outperform other unidimensional segregation measures (Larrabee Sonderlund et al., 2022; Krieger et al., 2017). Spatial, social, and economic marginalization and stigmatization of Black neighborhoods justifies policies and practices that lead to restricted access to resources and opportunity (like devaluation of housing and overall disinvestment (Uzzi et al., 2024)), limiting economic mobility (Larrabee Sonderlund et al., 2022), and potentially increasing reliance on underground economies for survival (Johnson and Bennett, 2017). Furthermore, unconstitutional and over policing of Black communities can worsen community violence through overly punitive police responses (Dawson et al., 2023) and reduced trust in the criminal legal system (Desmond et al., 2016), which can result in informal control measures that may lead to violence (Johnson and Bennett, 2017). Policies directed at addressing structural factors for violence and concentrated investment in the most vulnerable neighborhoods should be prioritized. This can include state and local policies to increase economic opportunities and reduce financial stress in the most disadvantaged neighborhoods.

We found the relationship between segregation and all assault hospital visits varied by vacant housing. In the most disadvantaged neighborhoods, higher levels of vacant housing were associated with more all assault visit rates, and this association decreased as neighborhood advantage increased. We also found vacant houses to be associated with higher all assault and firearm assault visit rates. This aligns with prior research (Branas et al., 2013; Roude et al., 2024). The presence of vacant houses does not cause violence. Rather, neighborhood physical signs of neglect, like the deteriorating facades of vacant houses, are a manifestation of structural factors like ongoing disinvestment and restricted access to economic capital and mobility (Bailey et al., 2017) that drive violence (Uzzi et al., 2023; Uzzi et al., 2024). Additionally, vacant properties can attract crime and serve as spaces where firearms or other weapons can be stored due to limited oversight and monitoring (Garvin et al., 2013; Branas et al., 2016). Interventions that remediate or demolish vacant properties have been associated with reductions in violent crimes, especially those related to firearms (Kondo et al., 2018b). Two potential mechanisms may explain these reductions: (1) the physical removal of these spaces limits opportunities for harboring crime, and (2) the perceived benefits of removing or rehabilitating vacant properties may promote positive street activities like increased social engagement and neighborhood connectedness, increasing eyes on the street and promoting social control to deter crime (Heinze et al., 2018; Aiyer et al., 2015).

Our study is not without limitations. It was cross-sectional and causality cannot be inferred. Our results are not generalizable since we only included five U.S. states. There is also the potential for unmeasured confounders that explain the relationship between segregation and assault hospital visits. The green space measures do not assess quality of green space. They only assess the percentage of each type of green space within each ZCTA. Different types of green space may be qualitatively different in disadvantaged neighborhoods compared to advantaged neighborhoods, or across neighborhoods with similar segregation patterns. Additionally, the park space variable includes data that were measured between 2016 and 2018 (Trust for Public Land, n.d.), which is not before our outcomes. Traditionally, it is not ideal to have exposures measured after the outcome; we do not expect park space to be significantly different in 2018 compared to 2015 because parks are difficult to develop. We only have the residential ZIP Code, not the ZIP Code where the assault occurred, which may be different. Last, we were unable to determine the propensity for residing in an advantaged versus disadvantaged neighborhood which may result in selection bias.

## 5 Conclusions

Our results highlight the potential mitigating role of forest space on the relationship between segregation and assault hospital visits, and underscores violence as a structural problem. This study advances current research by examining if the association between segregation and assault hospital visits vary by three green space measures and vacant housing. Overall, we found only forest space to be more protective in the most disadvantaged neighborhoods, with less association in advantaged neighborhoods; and vacant housing was a greater risk in the most disadvantaged neighborhoods.

## CRediT authorship contribution statement

**Lea A. Marineau:** Writing – review & editing, Writing – original draft, Methodology, Formal analysis, Conceptualization. **Kelly K. Jones:** Writing – review & editing, Data curation, Conceptualization. **Amanda K. Small:** Writing – review & editing. **Dennis W. Buckman:** Writing – review & editing, Methodology, Formal analysis. **Mudia Uzzi:** Writing – review & editing. **Melanie Sona:** Writing – review & editing, Data curation. **Erin I. Liedtke:** Writing – review & editing, Data curation. **Shannon N. Zenk:** Writing – review & editing, Supervision.

## Ethics approval

The National Institutes of Health Institutional Review Board (IRB) determined that this study is not human subjects research as defined by Department of Health and Human Services and Federal Drug Administration regulations. IRB review and approval was not required.

## Funding

This research was supported by the Intramural Research Program of the NIH, National Institute on Minority Health and Health Disparities.

## Declaration of competing interest

The authors declare that they have no known competing financial interests or personal relationships that could have appeared to influence the work reported in this paper.

## Data Availability

The authors do not have permission to share data.

## Appendix A Supplementary data

Appendix Table 1 Image 1
