# Supplementary material for: Racialized economic segregation, the built environment, and assault-related injury: Moderating role of green space and vacant housing
Source: Prev Med Rep. 2025 Jun 6;55:103128. doi: 10.1016/j.pmedr.2025.103128 (PMC12178919; doi:10.1016/j.pmedr.2025.103128)
Supplement: Supplementary file 1 — Appendix Table 1 [file mmc1.docx]

**Appendix Table 1: Matrix of correlations of vacant housing and green space variables for metropolitan ZIP Code Tabulation Areas in Arizona, Florida, Georgia, North Carolina, and New York**

| Variables | (1) | (2) | (3) | (4) |
| --- | --- | --- | --- | --- |
| (1) Vacant housing | 1.000 |  |  |  |
| (2) Developed open space | -0.245^***^ | 1.000 |  |  |
| (3) Park space | 0.072^***^ | -0.001 | 1.000 |  |
| (4) Forest space | 0.235^***^ | -0.296^***^ | -0.049^***^ | 1.000 |
|  | | | | |
| *** *P*<0.001; P-value was calculated by Pearson Correlation | | | | |
